# COVID-19–Related School Closures, United States, July 27, 2020–June 30, 2022

**DOI:** 10.3201/eid3001.231215

**Published:** 2024-01

**Authors:** Nicole Zviedrite, Ferdous Jahan, Sarah Moreland, Faruque Ahmed, Amra Uzicanin

**Affiliations:** Centers for Disease Control and Prevention, Atlanta, Georgia, USA (N. Zviedrite, F. Jahan, S. Moreland, F. Ahmed, A. Uzicanin);; Cherokee Nation Operational Solutions, LLC, Tulsa, Oklahoma, USA (F. Jahan);; Oak Ridge Institute for Science and Education, Oak Ridge, Tennessee, USA (S. Moreland)

**Keywords:** COVID-19, school closures, SARS-CoV-2, viruses, vaccine-preventable diseases, nonpharmaceutical interventions, community mitigation, United States, respiratory infections

## Abstract

As part of a multiyear project that monitored illness-related school closures, we conducted systematic daily online searches during July 27, 2020–June 30, 2022, to identify public announcements of COVID-19–related school closures (COVID-SCs) in the United States lasting >1 day. We explored the temporospatial patterns of COVID-SCs and analyzed associations between COVID-SCs and national COVID-19 surveillance data. COVID-SCs reflected national surveillance data: correlation was highest between COVID-SCs and both new PCR test positivity (correlation coefficient [r] = 0.73, 95% CI 0.56–0.84) and new cases (r = 0.72, 95% CI 0.54–0.83) during 2020–21 and with hospitalization rates among all ages (r = 0.81, 95% CI 0.67–0.89) during 2021–22. The numbers of reactive COVID-SCs during 2020–21 and 2021–22 greatly exceeded previously observed numbers of illness-related reactive school closures in the United States, notably being nearly 5-fold greater than reactive closures observed during the 2009 influenza (H1N1) pandemic.

Although unplanned school closures occur every year, outside of a pandemic, only a small minority (≈1%) are associated with infectious disease, whereas most are attributable to weather and natural disasters (*1*). However, the initial months (February–June 2020) of the COVID–19 pandemic in the United States led to unprecedented, nearly simultaneous, nationwide implementation of kindergarten through 12th grade (K–12) school closures throughout the United States as a part of a wider effort to slow virus transmission and reduce disease prevalence (*2*). In most communities, those early pandemic-related closures were implemented preemptively as a nonpharmaceutical intervention before community transmission was high. In contrast, the subsequent COVID–19-related school closures occurring in the 2020–21 and 2021–22 school years were predominantly reactive (i.e., occurring after infection had affected students, staff, or both).

As school reconvened for 2020–21 and 2021–22, schools and districts were faced with the challenge of providing in-person education and services during the ongoing pandemic. In a previous analysis, we described the transition to online learning that occurred during February–June 2020 after the COVID-19 related preemptive closures of schools and school districts (*2*). The subsequent 2 pandemic-affected school years (2020–21 and 2021–22) were characterized by the deployment of various education modalities, including education that was fully in-person, fully distance learning, or a hybrid model (*3*–*5*). In both years, among schools offering in-person learning (fully or hybrid), school closures continued to be implemented in response to local transmission dynamics and policies and to other consequences of the pandemic (e.g., vaccination of staff and students and side effects of vaccination, teacher and staff shortages, and pandemic-related mental health issues).

In this study, we describe trends in reactive COVID-19–related K–12 school closures (COVID-SCs) in the United States during July 27, 2020–June 30, 2022. We also analyze associations between COVID-SCs and national level COVID-19 epidemiologic surveillance data.

## Methods

### Data Collection

We conducted daily systematic online searches during July 27, 2020–June 30, 2022, to identify public announcements of unplanned, illness-related K–12 school closures in the United States. We conducted searches in Google and Google News by using the following terms: “school closed” and either “COVID,” “COVID-19,” or “coronavirus.” In addition, we used in a Google Alert the search string “(academy OR school OR district OR class) AND (close OR closing OR closure OR cancel OR cancelled) AND (coronavirus OR corona OR ‘COVID-19’ OR COVID OR ‘novel coronavirus’).” We also checked publicly available COVID-19–related school closure dashboards identified during those routine searches, including those published online by school districts, state and local education authorities, and private entities. We saved all school closure announcements as PDFs before data abstraction. We included for analysis only announcements mentioning COVID-19 as a reason for closure. Additional details on the search strategy and data abstraction processes have been published previously (*1*,*2*). We did not collect data during July 1–25, 2021, because this period coincided with school summer break in the United States. 

We classified fully in-person and hybrid learning modalities as open, and we classified modalities without in-person learning (i.e., fully distanced learning and closed) as closed. We defined unplanned closure as a transition from being open to being closed for in-person instruction for >1 day. If a school or district reopened for >1 day and then closed again, we counted the subsequent closure as a new occurrence of closure. For closures that spanned both unplanned and planned closure days, such as those contiguous with weekends or planned holidays, we counted only unplanned closure days. For closures for which a reopening date could not be identified after closure, we assumed the length of closure to be 1 day. Delay of in-person learning at the start of the school year, through closure or full distance-learning modalities, was not captured in this data collection. For each identified COVID-SC, we abstracted school or school district name, state, dates of closure and reopening, and reasons for closure as reported in the announcements.

### Contextualizing School Closures

To better understand the characteristics of schools and districts experiencing COVID-SCs, we matched each closure event to publicly available data downloaded from the National Center for Education Statistics (NCES) by using the respective NCES district or school identification (identified using district or school name and location) (*6*,*7*). Some districts and schools experienced multiple COVID-SC events during the study period and therefore appear >1 time in the resulting dataset. For each public school and public school district, we matched COVID-SC data with the respective year (i.e., 2020–21 or 2021–22) of NCES data, and for private schools, we matched COVID-SCs with the most recent year of data available (2019–20) in NCES. NCES data include information such as the number of schools per district, number of students and staff, number of students eligible in the federal free or reduced-price school lunch programs (only for public schools and public school districts), urbanicity, and grade span data. We excluded school districts with no schools; schools with no student enrollment; vocational, special education, and alternative schools with missing values for student enrollment; schools with prekindergarten, transitional kindergarten, or adult students only; and permanent distance learning–only schools (Appendix).

### Epidemiologic Data

We gathered publicly available COVID-19 surveillance data during July 27, 2020–June 30, 2022, including daily new cases and death counts (*8*), weekly hospitalization rates (*9*), and daily PCR positivity (*10*) for the duration of the study period. We reported hospitalization rates per epidemiologic week, and we calculated corresponding weekly figures for new cases, deaths, and PCR positivity at the national level. Epidemiologic weeks run Sunday through Saturday; epidemiologic week 1 was the first week to hold >4 days from the new calendar year.

### Data Analysis

We described characteristics of school closures according to the data abstracted from public announcements. We summarized specific reasons for COVID-SCs by grouping them into 12 non–mutually exclusive categories under 2 primary themes: transmission-related reasons and non–transmission-related reasons (Appendix). Transmission-related reasons included COVID-19 cases, suspected cases, increased student absenteeism, increased staff absenteeism, cluster or widespread transmission in the community, state or local guidance or mandate to close schools in response to COVID-19, cleaning or disinfecting school facilities, and other reasons related to COVID-19 mitigation, including testing, contact tracing, quarantine of students and staff, prevention of holiday-related surge, death of staff member, critical lack of community resources (e.g., contact tracers), and noncompliance with governor’s executive orders regarding nonpharmaceutical interventions. Non–transmission-related reasons included COVID-19 vaccinations, teacher or staff shortages, student or staff mental health, and other reasons associated with COVID-19, including staff protests of in-person learning, protests over mask policies, transportation issues specific to COVID-19, lack of resources specific to COVID-19, and work on the COVID-19 mitigation plan. We estimated in-person student-days lost because of COVID-SCs by multiplying the number of students per school by the respective number of unplanned closures days experienced.

We compared weekly patterns of COVID-SCs with COVID-19 epidemiologic surveillance data at the national level (new cases and deaths, hospitalization rates, and laboratory test positivity). We calculated Spearman rank correlations (r) and 95% CIs to evaluate these relationships (α = 0.05) during the school years. We excluded from analysis the final week of each calendar year because this week coincides with school winter break in the United States (Appendix). We calculated p values for the Spearman rank correlation on the basis of the Fisher Z-transformation. We conducted analysis by using SAS version 9.4 (SAS Institute Inc., https://www.sas.com) and visualized results by using Excel and Power BI (Microsoft, https://www.microsoft.com). In addition, we calculated in-person school days lost, the frequency and patterns of repeat closures of schools for COVID-19, and cumulative incidence of COVID-SCs by states, and we conducted bivariate and multivariable regression analyses (Appendix).

## Results

### School Year 2020–21

**Figure 1:**
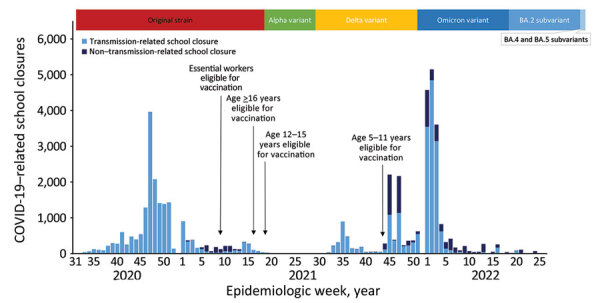
COVID-19–related school closures, dominant COVID-19 variants, and timing of vaccination availability, United States, July 27, 2020–June 30, 2022. School closure was defined as a transition from being open to being closed for in-person instruction excluding any scheduled days off; fully in-person and hybrid learning modalities were classified as open, and fully remote and closed were classified as closed. Transmission-related reasons were COVID-19 cases, suspected cases, increased student absenteeism, increased staff absenteeism, cluster or widespread transmission in the community, state or local guidance or mandate to close schools in response to COVID-19, to clean or disinfect school facilities, and other. Non–transmission-related reasons were COVID-19 vaccinations and side effects of vaccination of staff or students, teacher or staff shortage, for student or staff mental health, and other reasons associated with COVID-19. Timeline of COVID-19 variants derived from Centers for Disease Control and Prevention Museum COVID-19 Timeline (*11*) and defined as the point at which a variant accounted for the largest proportion of cases. Emergency Use Authorization by the Food and Drug Administration authorized COVID-19 vaccination for teachers and staff as part of the essential workforce on March 2, 2021, and all persons >16 years of age on April 19, 2021 (*12*). Advisory Committee on Immunization Practices recommended COVID-19 vaccination for persons 12–15 years of age on May 12, 2021, and for persons 5–11 years of age on November 2, 2021 (*11*).

During July 27, 2020–June 30, 2021, a total of 16,890 unique schools experienced an estimated 19,273 COVID-SCs (Table 1). Approximately 75% closed as part of districtwide closures. More than 11 million students were affected, and >159 million in-person student-days were lost. Most COVID-SCs were observed in the first half of the school year (August–December), and they peaked in epidemiologic week 47, the week before the US Thanksgiving holiday (Figure 1). The median number of in-person schools days lost per closure nationally was 10 days (interquartile range [IQR] 3–23 days) (Appendix Figure 1); however, this figure reached >20 days in 7 states (California, Colorado, Illinois, Indiana, Kentucky, Minnesota, and Nevada) (Appendix Figure 2). Most schools experiencing COVID-SCs experienced only 1 COVID-SC during the school year (Appendix Table 1). However, 2,036 schools (12.1%) experienced 2–7 COVID-SCs during the school year; 2 was most common (1,756 schools [86.2%]).

**Table 1:** Characteristics of COVID-19–associated school closures, by school year, United States, July 27, 2020–June 30, 2022*

Characteristics of COVID-19–associated school closures	Total	School year†	
		2020–21	2021–22
No. school closures‡	10,884	6,322 (58.1)	4,562 (41.9)
Districtwide	3,443 (31.6)	1,528 (24.2)	1,915 (42.0)
Individual school	7,441 (68.4)	4,794 (75.8)	2,647 (58.0)
Total estimated no. unique schools closed	36,761	16,890 (45.9)	19,871 (54.1)
Total estimated no. closed schools§¶	45,180	19,273 (42.7)	25,907 (57.3)
Closure type			
Districtwide	37,739 (83.5)	14,479 (75.1)	23,260 (89.8)
Individual school	7,441 (16.5)	4,794 (24.9)	2,647 (10.2)
School type			
Public	44,463 (98.4)	18,620 (96.6)	25,843 (99.8)
Private	717 (1.6)	653 (3.4)	64 (0.3)
School grade level§			
Elementary school: K–5th grade	18,273 (40.4)	7,701 (40.0)	10,572 (40.8)
Elementary–middle school: K–8th grade	8,241 (18.2)	3,037 (15.8)	5,204 (20.1)
Elementary–high school: K–12th grade	1,107 (2.5)	461 (2.4)	646 (2.5)
Middle school: 6–8th grade	6,405 (14.2)	2,751 (14.3)	3,654 (14.1)
Middle–high school: 6–12th grade	2,137 (4.7)	969 (5.0)	1,168 (4.5)
High school: 9–12th grade	8,587 (19.0)	4,048 (21.0)	4,539 (17.5)
Not specified	430 (1.0)	306 (1.6)	124 (0.5)
Season			
Fall: Sep–Nov	18,298 (40.5)	11,660 (60.5)	6,638 (25.6)
Winter: Dec–Feb	22,651 (50.1)	5,694 (29.5)	16,957 (65.5)
Spring: Mar–May	2,818 (6.2)	1,642 (8.5)	1,176 (4.5)
Summer: Jun–Aug	1,413 (3.1)	277 (1.4)	1,136 (4.4)
Urbanicity			
City	17,689 (39.2)	6,734 (34.9)	10,955 (42.3)
Suburban	13,609 (30.1)	6,116 (31.7)	7,493 (28.9)
Town	4,429 (9.8)	1,959 (10.2)	2,470 (9.5)
Rural	9,098 (20.1)	4,181 (21.7)	4,917 (19.9)
Not specified	355 (0.8)	283 (1.5)	72 (0.3)
HHS region#			
HHS 1	1,906 (4.2)	1,301 (6.8)	605 (2.3)
HHS 2	4,556 (10.1)	3,146 (16.3)	1,410 (5.4)
HHS 3	5,642 (12.5)	2,886 (15.0)	2,756 (10.6)
HHS 4	9,399 (20.8)	3,978 (20.6)	5,421 (20.9)
HHS 5	9,646 (21.4)	3,627 (18.8)	6,019 (23.1)
HHS 6	5,103 (11.3)	1,050 (5.5)	4,053 (15.6)
HHS 7	2,217 (4.9)	588 (3.1)	1,634 (6.3)
HHS 8	2,493 (5.5)	872 (4.5)	1,621 (6.3)
HHS 9	2,334 (5.2)	1,453 (7.5)	881 (3.4)
HHS 10	1,879 (4.2)	372 (1.9)	1,507 (5.8)
No. students affected§**	25,837,466	11,232,072 (43.5)	14,605,394 (56.5)
No. teachers affected§††	1,710,459	752,264 (44.0)	958,195 (56.0)
% Students eligible for free or reduced-price lunch,§‡‡ median (IQR)	57.2 (33.2–83.2)	52.0 (28.8–79.1)	60.9 (37.0–85.7)
No. in-person student-days lost§§	205,689,158	159,968,778 (77.8)	45,720,380 (22.2)
No. unplanned closure days,¶¶ median (IQR)	4 (1–10)	10 (3–23)	2 (1–4)

*Values are no. (%) except as indicated. HHS, US Department of Health and Human Services; ID, identification; IQR, interquartile range; K, kindergarten; NCES, National Center for Education Statistics; PSS, the Private School Universe Survey. †School years: 2020–21 (July 27, 2020–June 30, 2021), 2021–22 (August 1, 2021–June 30, 2022). ‡School closures were defined as a transition from being opened to being closed for in-person instruction excluding any scheduled days off; fully in-person and hybrid learning modalities were classified as open, and fully remote and closed were classified as closed. Closure events were documented at either the district-level or the individual school level on the basis of the source and scope of the closure decision as reported in the announcements. §Schools were counted once for each time they were part of a school closure event at either the district-level or school level. ¶Number of schools closed in district-wide closures, total number of students, total number of teachers, number of students eligible for federal free or reduced-priced lunch, and number of schools by urbanicity and grade levels were estimated by matching the public school district ID or public school ID with the district or school data with the respective year, as obtained from NCES and private school ID with the 2019–20 private school data, as obtained from PSS. Because of missing information on urbanicity in 2021–22 NCES public school data, the information on urbanicity for the 2021–22 school year public school closures was obtained from the 2020–21 NCES public school data (*6**,**7*). School NCES ID was not found for 168 schools (124 for 2020–21 school year and 44 for 2021–22 school year), which were categorized as public or private by manual search on the basis of the location of schools in the school closure announcements. #HHS regions defined at https://www.hhs.gov/about/agencies/regional-offices/index.html. **Students were counted once for each school closure event. The number of students was missing for 562 schools (public, 325; private, 237). ††Teachers were counted once for each school closure event. Part-time teaching positions were reported as a fraction of 1 full-time position. The number of teachers was missing for 1,041 schools (public, 804; private, 237). ‡‡Data only for public schools. §§In-person student-days lost estimated by multiplying the number of students per school by the number of unplanned closure days. ¶¶2,075 schools did not have reopening dates and defaulted to a 1-day closure; among these, 1,996 (96%) were during the 2020–21 school year.

Among the announcements reporting reasons for closure, the most common reasons were having positive COVID-19 cases in the school (47.4% of district-level and 58.5% of school-level events) and clusters or widespread transmission in the community (47.8% of district-level and 32.5% of school-level events) (Table 2). In addition, state and local mandates to close schools in response to COVID-19 accounted for >20% of closures. When analysis was restricted to the 7 states with the highest median closure lengths (California, Colorado, Illinois, Indiana, Kentucky, Minnesota, and Nevada), clusters or widespread transmission in the community accounted for greater proportions of district-level (83.1%) and school-level (55.4%) closure events (Appendix Table 2). Similarly, closure events attributable to state and local mandates were proportionally higher in these states at the district level (28.8%) and school level (30.7%) than in the nation as a whole (Appendix Table 2). Mandates were issued either as part of an ongoing policy triggered by reaching a COVID-19 threshold, such as the positivity rate of testing (*13*), or in response to local surges in cases (*14*,*15*). Although few COVID-SCs were attributed to non–transmission-related reasons, such as vaccination of staff and students or nonspecific teacher shortages attributed to the pandemic, nearly 30% of COVID-SCs during epidemiologic weeks 6–11 of 2021 were attributable to vaccination of staff and side effects of vaccination (Figure 1).

**Table 2:** Reasons for COVID-19–related K–12 school closure, United States, July 27, 2020–June 30, 2022*

Reasons for school closure decision stated in closure announcement†	Total			2020–21‡			2021–22‡	
	District	School		District	School		District	School
COVID-19–related closure	3,443	7,441		1,528	4,794		1,915	2,647
COVID-19 only	85 (2.5)	123 (1.7)		43 (2.8)	76 (1.6)		42 (2.2)	49 (1.9)
COVID-19 and specific reasons	3,358 (97.5)	7,318(98.3)		1,485 (97.2)	4,718 (98.4)		1,873 (97.8)	2,598 (98.1)
Transmission-related reasons								
Positive case	1,845 (53.6)	4,511 (60.6)		724 (47.4)	2,829 (59.0)		1,121 (58.5)	1,682 (61.5)
In student	758 (22.0)	1,412 (19.0)		233 (15.2)	651 (13.6)		525 (27.4)	761 (28.7)
In staff member	763 (22.2)	1,359 (18.3)		226 (14.8)	575 (12.0)		537 (28.0)	784 (29.6)
In household member§	15 (0.4)	24 (0.3)		11 (0.7)	12 (0.3)		4 (0.2)	12 (0.5)
In visitor	2 (0.1)	0		2 (0.1)	0		0	0
Suspected case	78 (2.3)	224 (3.0)		64 (4.2)	173 (3.6)		14 (0.7)	51 (1.9)
In student	23 (0.7)	70 (0.9)		21 (1.4)	48 (1.0)		2 (0.1)	22 (0.8)
In staff member	33 (1.0)	71 (1.0)		27 (1.8)	53 (1.1)		6 (0.3)	18 (0.7)
In household member§	0	5 (0.1)		0	5 (0.1)		0	0
Increased student absenteeism	515 (15.0)	732 (9.8)		56 (3.7)	132 (2.8)		459 (24.0)	600 (31.3)
Increased staff absenteeism	1,022 (29.7)	1,955 (26.3)		183 (12.0)	536 (11.2)		839 (43.8)	1,419 (53.6)
Cluster or widespread transmission¶	1,096 (31.8)	1,918 (25.8)		727 (47.8)	1,556 (32.5)		369 (19.3)	362 (13.7)
State or local guidance or mandate	348 (10.1)	1,146 (15.4)		318 (20.8)	1,091 (22.8)		30 (1.6)	55 (2.1)
To clean or disinfect#	240 (7.0)	682 (9.2)		102 (6.7)	562 (11.7)		138 (7.2)	120 (4.5)
Other**	356 (10.3)	750 (10.1)		146 (9.6)	532 (11.1)		210 (11.0)	218 (8.2)
Non–transmission-related reasons								
Vaccination of staff or students	83 (2.4)	56 (0.8)		66 (4.3)	49 (1.0)		17 (0.9)	7 (0.3)
Side effects of vaccination	9 (0.3)	18 (0.2)		9 (0.6)	18 (0.4)		0	0
Teacher shortage	135 (3.9)	158 (2.1)		20 (1.3)	26 (0.5)		115 (6.0)	132 (5.0)
Mental health	158 (4.6)	48 (0.6)		0	0		158 (8.3)	48 (1.8)
Other††	17 (0.5)	32 (0.4)		0	10 (0.2)		17 (0.9)	22(0.8)

*Values are no. (%) except as indicated. †Reasons are recorded as stated in the school closure announcements. Categories are not mutually exclusive because a closure announcement may attribute the closure to >1 factor, there may be >1 announcement that contributes the closure to different factors, or both. ‡School year: 2020–21 (July 27, 2020–June 30, 2021), 2021–22 (August 1, 2021–June 30, 2022). §Of student or staff. ¶In the community. #Classrooms, buildings, and facilities **Other reasons were contact tracing, quarantine of students and staff, prevention of holiday-related surge, precautionary measure as a concern of community spread because of union wanting work stoppage, death of staff member, inability to find substitute teachers, transportation issues, critical lack of community resources (including contact tracers), testing, out of an abundance of caution, influenza or other respiratory virus-related or enteric virus-related illnesses, internet outage, facility issues, and noncompliance with governor’s executive orders regarding nonpharmaceutical interventions. ††Other reasons were staff protesting in-person learning, protest over mask policy, transportation issue, lack of resources, allowing time for testing, and work on the COVID-19 mitigation plan.

**Figure 2:**
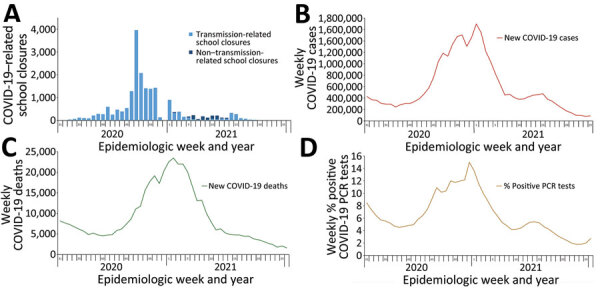
COVID-19–related school closures and COVID-related cases, deaths, and PCR positivity by school year, United States, July 27, 2020–June 30, 2021. School closure, transmission-related reasons, and non–transmission-related reasons are defined in the Figure 1 legend. Data on COVID-19 cases and COVID-19–associated deaths available from Centers for Disease Control and Prevention (*8*). PCR positivity was calculated from the number of new positive results divided by the total number of new results reported. Data on PCR testing were available from US Department of Health and Human Services (*10*). School year: 2020–21 (July 27, 2020–June 30, 2021).

**Figure 3:**
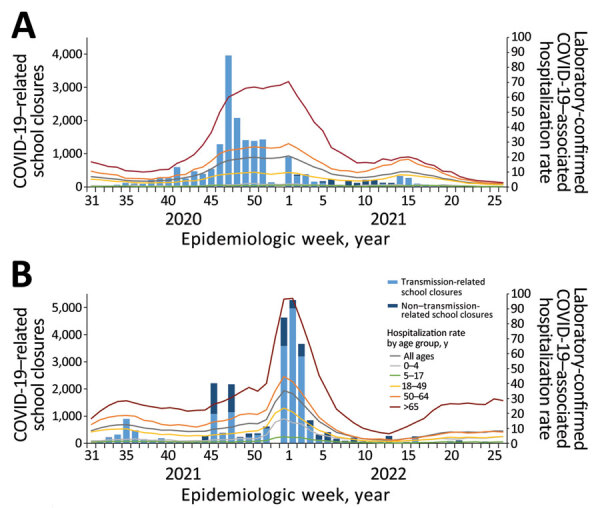
COVID-19–related school closures and laboratory-confirmed COVID-19–related hospitalizations by age group, United States, July 27, 2020–June 30, 2022. A) School year 2020–21 (July 27, 2020–June 30, 2021); B) school year 2021–22 (August 1, 2021–June 30, 2022). School closure, transmission-related reasons, and non–transmission-related reasons are defined in the Figure 1 legend. Data on laboratory-confirmed COVID-19–associated hospitalizations available from COVID-NET (*9*).

Transmission-related COVID-SCs were strongly correlated with weekly COVID-19 testing positivity rates (r = 0.73, 95% CI 0.56–0.84) and with new COVID-19 cases (r = 0.72, 95% CI 0.54–0.83) (Table 3). Transmission-related COVID-SCs were moderately correlated with both new COVID-19 deaths by week (r = 0.51, 95% CI 0.25–0.69) and weekly laboratory confirmed COVID-19–associated hospitalization rates for all ages (r = 0.64, 95% CI 0.42–0.78) (Table 4). Age-specific correlation with hospitalization rates varied; the 5–17-year age group, which aligns with the K–12 student population, had the weakest correlation (r = 0.37, 95% CI 0.09–0.60), and correlations strengthened for each subsequent older age group (Table 4). The peak in weekly COVID-SCs preceded the peaks in COVID-19 disease surveillance indicators (new cases, new deaths, percentage positive PCR tests, and hospitalization rates) by roughly 6–8 weeks (Figure 2, panels A–D; Figure 3, panel A).

**Table 3:** Weekly correlation between COVID-19–related school closures and COVID-19–related cases, deaths, and PCR positivity, by school year, United States, July 27, 2020–June 30, 2022*

School closure type	Weeks 52–53			Weeks considered as winter school break†				
				Weeks 52–53 or weeks 51–52			Weeks 53–1 or weeks 52–1	
r (95% CI)	p value	r (95% CI)	p value	r (95% CI)	p value
Transmission-related school closures‡								
COVID-19 cases§								
2020–21	0.721 (0.544–0.832)	<0.001		0.728 (0.552–0.837)	<0.001		0.707 (0.521–0.824)	<0.001
2021–22	0.609 (0.384–0.760)	<0.001		0.591 (0.358–0.750)	<0.001		0.583 (0.346–0.744)	<0.001
COVID-19 deaths§								
2020–21	0.507 (0.255–0.688)	<0.001		0.510 (0.256–0.692)	<0.001		0.482 (0.221–0.673)	<0.001
2021–22	0.580 (0.346–0.741)	<0.001		0.592 (0.359–0.750)	<0.001		0.571 (0.331–0.736)	<0.001
PCR positivity¶								
2020–21	0.734 (0.563–0.840)	<0.001		0.748 (0.581–0.850)	<0.001		0.720 (0.540–0.832)	<0.001
2021–22	0.280 (−0.011–0.523)	0.056		0.245 (−0.052–0.497)	0.102		0.232 (−0.065–0.487)	0.122
Total school closures#								
COVID-19 cases§								
2020–21	0.772 (0.620–0.864)	<0.001		0.790 (0.645–0.876)	<0.001		0.761 (0.601–0.858)	<0.001
2021–22	0.511 (0.257–0.693)	<0.001		0.486 (0.223–0.678)	<0.001		0.478 (0.213–0.672)	0.001
COVID-19 deaths§								
2020–21	0.577 (0.345–0.737)	<0.001		0.589 (0.358–0.747)	<0.001		0.557 (0.316–0.725)	<0.001
2021–22	0.596 (0.367–0.751)	<0.001		0.610 (0.382–0.762)	<0.001		0.587 (0.353–0.747)	<0.001
PCR positivity¶								
2020–21	0.695 (0.506–0.815)	<0.001		0.721 (0.542–0.833)	<0.001		0.679 (0.480–0.806)	<0.001
2021–22	0.190 (−0.105–0.451)	0.202		0.149 (−0.149–0.420)	0.325		0.136 (−0.162–0.409)	0.369

*School closure is defined as a transition from being open to being closed for in-person instruction excluding any scheduled days off; fully in-person and hybrid learning modalities are classified as open, and fully remote and closed are classified as closed. School year: 2020–21 (July 27, 2020–June 30, 2021), 2021–22 (August 1, 2021–June 30, 2022). Spearman rank correlation (r) was used to evaluate the relationship between COVID-19–associated cases, deaths, and PCR positivity (epidemiologic weeks 31–26). †School winter break was excluded from the analysis. The break is understood to be ≈2 weeks in length; however, the start and end dates vary by school and district. Winter breaks consistently overlap on the last week of the year, which was epidemiologic week 53 in 2020 and epidemiologic week 52 in 2021. In 2020, we calculated correlations excluding winter break at epidemiologic week 53, weeks 52–53, and weeks 53–1. In 2021, correlations were calculated excluding winter break at epidemiologic week 52, weeks 51–52, and weeks 52–1. ‡Transmission-related reasons were COVID-19 cases, suspected cases, increased student absenteeism, increased staff absenteeism, cluster or widespread transmission in the community, state or local guidance or mandate to close schools in response to COVID-19, to clean or disinfect school facilities, and other. §Data on COVID-19 cases and COVID-19–associated deaths available from Centers for Disease Control and Prevention (*8*). ¶PCR positivity was calculated from the number of new positive results divided by the total number of new results reported. Data on PCR testing were available from US Department of Health and Human Services (*10*). #Total school closures were both transmission and non–transmission-related school closures. Transmission-related reasons were COVID-19 cases, suspected cases, increased student absenteeism, increased staff absenteeism, cluster or widespread transmission in the community, state or local guidance or mandate to close schools in response to COVID-19, to clean or disinfect school facilities, and other. Non–transmission-related reasons were COVID-19 vaccinations and side effects of vaccination of staff or students, teacher or staff shortage, for student or staff mental health, and other reasons associated with COVID-19.

**Table 4:** Weekly correlation between COVID-19–related school closures and laboratory-confirmed COVID-19–associated hospitalizations, by school year and age group, United States, July 27, 2020–June 30, 2022*

School closure type	Weeks 52–53			Weeks considered as winter school break†				
				Weeks 52–53 or weeks 51–52			Weeks 53–1 or weeks 52–1	
	r (95% CI)	P value		r (95% CI)	p value		r (95% CI)	p value
Transmission-related school closures‡								
2020–21								
All ages	0.639 (0.420–0.783)	<0.001		0.650 (0.431–0.791)	<0.001		0.618 (0.387–0.770)	<0.001
0–4 y	0.403 (0.119–0.620)	0.006		0.406 (0.120–0.625)	0.006		0.367 (0.074–0.596)	0.014
5–17 y	0.373 (0.085–0.598)	0.011		0.378 (0.087–0.604)	0.011		0.339 (0.043–0.575)	0.024
18–49 y	0.548 (0.298–0.722)	<0.001		0.548 (0.294–0.723)	<0.001		0.520 (0.258–0.705)	<0.001
50–64 y	0.622 (0.396–0.771)	<0.001		0.630 (0.404–0.778)	<0.001		0.599 (0.362–0.758)	<0.001
>65 y	0.669 (0.461–0.802)	<0.001		0.685 (0.480–0.813)	<0.001		0.650 (0.431–0.791)	<0.001
2021–22								
All ages	0.812 (0.667–0.894)	<0.001		0.803 (0.650–0.890)	<0.001		0.798 (0.641–0.886)	<0.001
0–4 y	0.357 (0.051–0.596)	0.021		0.321 (0.006–0.572)	0.043		0.308 (−0.008–0.563)	0.053
5–17 y	0.687 (0.474–0.818)	<0.001		0.676 (0.454–0.813)	<0.001		0.663 (0.436–0.805)	<0.001
18–49 y	0.761 (0.586–0.864)	<0.001		0.750 (0.565–0.858)	<0.001		0.743 (0.555–0.854)	<0.001
50–64 y	0.827 (0.692–0.903)	<0.001		0.820 (0.677–0.899)	<0.001		0.814 (0.668–0.896)	<0.001
>65 y	0.700 (0.494–0.827)	<0.001		0.685 (0.468–0.818)	<0.001		0.677 (0.457–0.814)	<0.001
Total school closures§								
2020–21								
All ages	0.651 (0.437–0.791)	<0.001		0.675 (0.467–0.807)	<0.001		0.631 (0.405–0.779)	<0.001
0–4 y	0.386 (0.100–0.608)	0.008		0.401 (0.114–0.621)	0.007		0.349 (0.054–0.582)	0.020
5–17 y	0.351 (0.060–0.581)	0.018		0.368 (0.075–0.596)	0.014		0.315 (0.016–0.557)	0.037
18–49 y	0.533 (0.278–0.712)	<0.001		0.5444 (0.288–0.721)	<0.001		0.503 (0.237–0.693)	<0.001
50–64 y	0.630 (0.407–0.776)	<0.001		0.651 (0.432–0.791)	<0.001		0.608 (0.373–0.763)	<0.001
>65 y	0.720 (0.535–0.834)	<0.001		0.752 (0.580–0.855)	<0.001		0.705 (0.510–0.826)	<0.001
2021–22								
All ages	0.708 (0.518–0.827)	<0.001		0.693 (0.492–0.818)	<0.001		0.689 (0.486–0.815)	<0.001
0–4 y	0.470 (0.200–0.668)	0.001		0.442 (0.162–0.650)	0.002		0.433 (0.152–0.644)	0.003
5–17 y	0.646 (0.429–0.787)	<0.001		0.630 (0.404–0.778)	<0.001		0.621 (0.391–0.772)	<0.001
18–49 y	0.645 (0.427–0.786)	<0.001		0.625 (0.397–0.775)	<0.001		0.620 (0.390–0.771)	<0.001
50–64 y	0.723 (0.539–0.836)	<0.001		0.708 (0.514–0.828)	<0.001		0.704 (0.508–0.825)	<0.001
>65 y	0.677 (0.472–0.806)	<0.001		0.659 (0.443–0.797)	<0.001		0.654 (0.437–0.793)	<0.001

*School closure is defined as a transition from being open to being closed for in-person instruction excluding any scheduled days off; fully in-person and hybrid learning modalities are classified as open, and fully remote and closed are classified as closed. School year: 2020–21 (July 27, 2020–June 30, 2021), 2021–22 (August 1, 2021–June 30, 2022). Data on laboratory-confirmed COVID-19–associated hospitalizations was available from COVID-NET (*9*). Spearman rank correlation (r) was used to evaluate the relationship between COVID-19–associated cases, deaths, and PCR-positivity (epidemiologic weeks 31–26). †School winter break was excluded from the analysis. The break is understood to be ≈2 weeks in length; however, the start and end dates vary by school and district. Winter breaks consistently overlap on the last week of the year, which was epidemiologic week 53 in 2020 and epidemiologic week 52 in 2021. In 2020, we calculated correlations excluding winter break at epidemiologic week 53, weeks 52–53, and weeks 53–1. In 2021, correlations were calculated excluding winter break at epidemiologic week 52, weeks 51–52, and weeks 52–1. ‡Transmission-related reasons were COVID-19 cases, suspected cases, increased student absenteeism, increased staff absenteeism, cluster or widespread transmission in the community, state or local guidance or mandate to close schools in response to COVID-19, to clean or disinfect school facilities, and other. §Total school closures were both transmission and non–transmission-related school closures. Transmission-related reasons were COVID-19 cases, suspected cases, increased student absenteeism, increased staff absenteeism, cluster or widespread transmission in the community, state or local guidance or mandate to close schools in response to COVID-19, to clean or disinfect school facilities, and other. Non–transmission-related reasons were COVID-19 vaccinations and side effects of vaccination of staff or students, teacher or staff shortage, for student or staff mental health, and other reasons associated with COVID-19.

### School Year 2021–22

During August 1, 2021–June 30, 2022, more than 14.6 million students in the United States were affected by an estimated 25,907 COVID-SCs (Table 1). Among unique schools that experienced COVID-SCs, most closed once (77.5%), whereas >20% closed 2–8 times (Appendix Table 1). Most closures occurred in the first 3 weeks of 2022, peaking at epidemiologic week 2 (Figure 1). The median number of in-person school days lost per closure was 2 days (IQR 1–4 days) (Appendix Figure 1).

Closures occurred in all 50 states and the District of Columbia; >1,000 closures occurred in each of 7 states (Georgia, Illinois, Missouri, North Carolina, Ohio, Tennessee, and Texas) (Appendix Figure 3, panel A), accounting for more than one third of all COVID-SCs observed during the school year. COVID-SCs were experiences by more than half of the schools in Alabama (51.6%), Nevada (51.3%), and Oregon (51.2%) and by 40%–50% of schools in 8 additional states Colorado, Kentucky, Maryland, Nebraska, Oklahoma, Tennessee, Utah, and Virginia) (Appendix Figure 3, panel B).

Most COVID-SC events were attributed to positive cases in the schools (58.5% of district-level and 61.5% of school-level events) (Table 2). Non–transmission-related reasons accounted for a greater number of COVID-SCs than in the prior school year, particularly those attributable to long-term teacher or staff shortages (e.g., shortages related to hiring and retention challenges rather than directly linked to current disease transmission).

**Figure 4:**
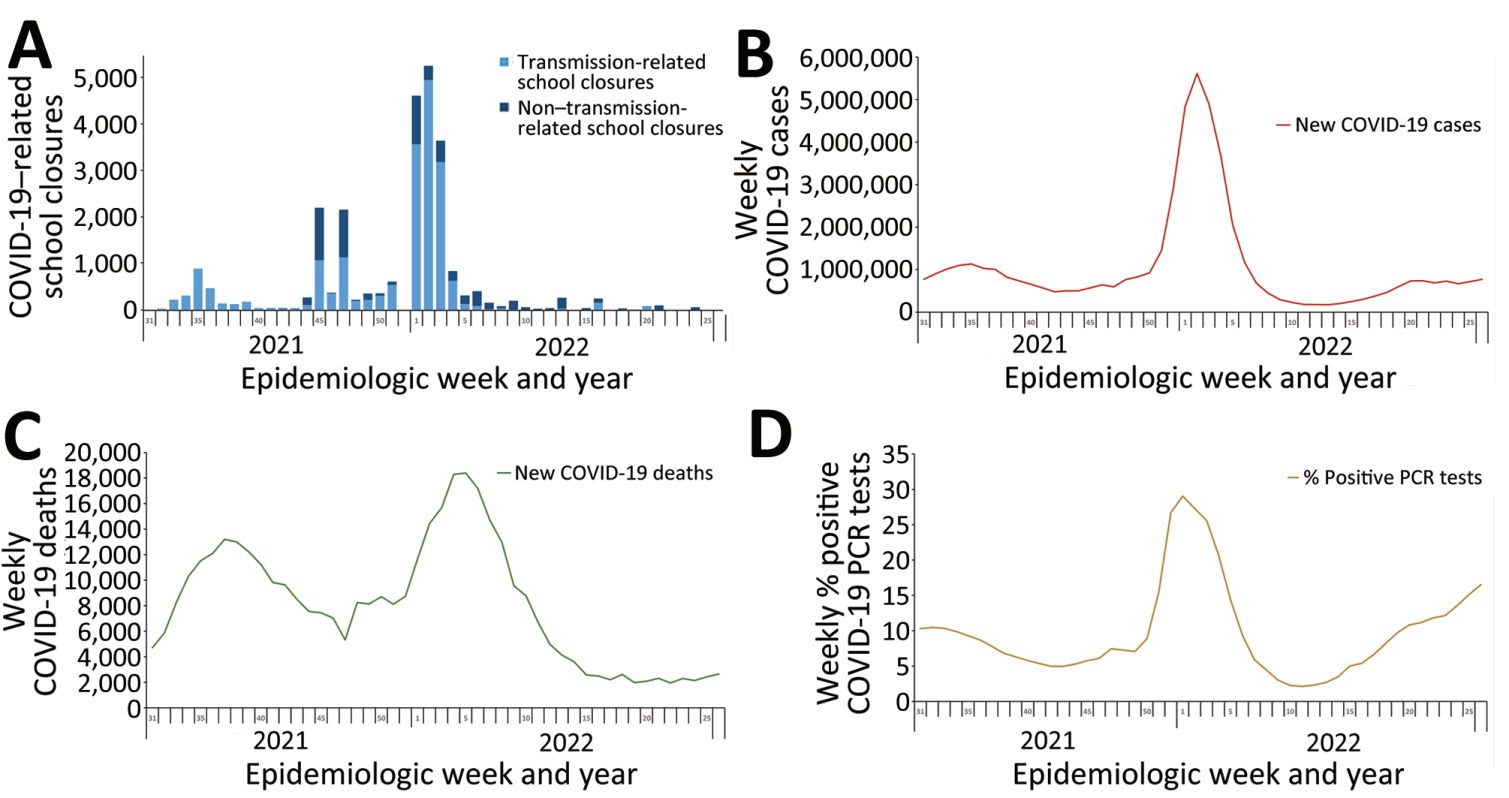
COVID-19–related school closures and COVID-related cases, deaths, and PCR positivity by school year, United States, August 1, 2021–June 30, 2022. School closure, transmission-related reasons, and non–transmission-related reasons are defined in the Figure 1 legend. Data on COVID-19 cases and COVID-19–associated deaths available from Centers for Disease Control and Prevention (*8*). PCR positivity was calculated from the number of new positive results divided by the total number of new results reported. Data on PCR testing were available from US Department of Health and Human Services (*10*). School year: 2021–22 (August 1, 2021–June 30, 2022).

Transmission-related COVID-SCs were strongly correlated with weekly hospitalization rates for all ages (r = 0.81, 95% CI 0.67–0.89), among which correlation was moderate for the 5–17-year age group (r = 0.69, 95% CI 0.47–0.82) and strong for the 3 adult age groups (Table 4). Transmission-related COVID-SCs were moderately correlated with new COVID-19 cases (r = 0.61, 95% CI 0.38–0.76) and new COVID-19 deaths (r = 0.58, 95% CI 0.35–0.74), whereas we observed no significant correlation for percentage positive COVID-19 PCR tests (Table 3). The peak in weekly COVID-SCs occurred within 1–2 weeks of the peaks in weekly new COVID-19 cases, percentage PCR positivity, and hospitalization rates and preceded the peak in weekly new COVID-19 deaths by 3 weeks (Figure 4, panels A–D; Figure 3, panel B).

## Discussion

Our study describes COVID-19 school closures in the United States as school systems and communities grappled with ongoing disease transmission during a rapidly evolving pandemic. The COVID-SCs we analyzed reflect SARS-CoV-2 spread among school-aged children and staff, as demonstrated by the correlation between COVID-SCs and COVID-19 epidemiologic surveillance. The large increase in illness-related closures during the 2020–21 and 2021–22 school years, which was nearly 5-fold higher than those observed during severe influenza seasons, including the 2009 influenza (H1N1) pandemic (*16*) and subsequent moderate and severe influenza seasons (e.g., 2017–18, 2018–19, and 2019–20), was nearly fully attributed to COVID-19 (Zviedrite et al., unpub. data, https://doi.org/10.1101/2023.08.28.23294732). This increase probably reflected both ongoing transmission of SARS-CoV-2 and the greater clinical severity of COVID-19 infection among children compared with influenza, as demonstrated by both higher rates of hospitalization and higher rates of intensive care unit admission among those <18 years of age (*17*,*18*).

The most frequently documented reason for closure in both school years was COVID-19 cases in the school or school district. School year 2020–21 also had a significant number of closures attributable to increased community transmission and to state or local mandates to close schools in response to COVID-19, consistent with the changing incidence of COVID-19 among both the general population and children during the study period (*8*,*9*). COVID-SCs attributable to teacher absenteeism (because of illness in themselves or others) and teacher or staff shortages were more common in 2021–22 than in SY 2020–21. Although teacher shortages also were reported before the pandemic, they were exacerbated by the pandemic (*19*,*20*).

In 2020–21, the number of schools open to in-person education varied throughout the semester; some schools were teaching in-person, some relied solely on distance learning, some used hybrid or mixed methods, and others moved among different modalities in response to disease transmission and related guidance throughout the school year (*5*). COVID-19 transmission during 2020–21 was primarily dominated by the ancestral strain of SARS-CoV-2, which would only later be supplanted by new variants as the predominating virus in circulation (*21*,*22*). Most COVID-SCs in 2020–21 occurred in the first semester (August– December 2020) before peaks in COVID-19 epidemiologic surveillance data; the bulk occurred in the 2 weeks leading up to the 2020 Thanksgiving holiday break. The wording of reasons for closure as abstracted from some school closure announcements suggested that, amidst increasing transmission, schools and school districts were trying to take advantage of a planned break and lengthen the total time outside of the classroom by closing schools early. Despite recommendations from the Centers for Disease Control and Prevention against travel during the holiday (*23*), travel during Thanksgiving week of 2020 (November 22–28, 2020) was at its highest since the start of the pandemic 8 months prior (*24*,*25*). During this time of increased social gatherings and movement, many schools that closed before Thanksgiving chose to stay shuttered until after the subsequent winter break (*26*–*28*). Annual (school year) COVID-19 peaks in the epidemiologic surveillance data occurred in January 2021. After Emergency Use Authorization was issued by the Food and Drug Administration for the first COVID-19 vaccine in December 2020 (*29*), teachers and staff became eligible for vaccination as part of the essential workforce on March 2, 2021, and all those >16 years of age became eligible for vaccination on April 19, 2021 (*12*). Subsequently, the succeeding Alpha variant began to predominate in early April 2021 (*11*,*22*), holding that position until June as the number of COVID-SCs slowed and remained comparably low.

Thereafter, the Delta variant, which was more transmissible and more severe than both the ancestral strain or Alpha variant (*30*,*31*), predominated through the start of school year 2021–22 until mid-December (*22*). During that period, the Food and Drug Administration’s Emergency Use Authorizations for COVID-19 vaccines were extended to persons 12–15 years of age in May 2021 and to those 5–11 years of age in October 2021 (*11*), whereby all K–12 students, teachers, and staff were eligible for vaccination during the first half of the 2021–22 school year. Prior to the start of that school year, nearly 90% of teachers were vaccinated nationally (*32*). Expanded vaccine eligibility and high uptake among teachers and staff coincided with a return to in-person learning for nearly all schools in the United States in 2021–22 (*3*,*5*). In that school year, according to the epidemiologic surveillance data, COVID-19 peaked in January 2022 amid the domination of the Omicron variant, thus far characterized as the fastest-spreading variant (*33*), and was matched with the highest weekly counts of COVID-SCs. Although we observed lower correlation of COVID-SCs with COVID-19 cases and percentage PCR positivity in 2021–22 than in the previous year, correlations with new deaths and hospitalization rates were both higher. Strong correlation between COVID-SCs and hospitalization rates may suggest that COVID-SCs are not only associated with disease prevalence but also with the severity of the dominant circulating variant.

Various prevention measures were implemented in schools and districts to prevent the spread of SARS-CoV-2 (*34*) and thereby reduce the number of COVID-SCs. However, disparities in their implementation have been observed across locales and school poverty levels (*34*). Infection-prevention measures include nonpharmaceutical interventions that can be rapidly implemented in schools, such as masking, social distancing, and quarantining, all of which have previously been shown to be effective at slowing influenza transmission in community congregate settings (*35*); at least 1 study documented that use of face masks reduces SARS-CoV-2 infection incidence in K–12 schools (*36*).

One limitation of our study is that reports are limited to publicly available data, and some closures may have been missed depending on how they were reported. In addition, data may be incomplete for various reasons, including delays in identifying public announcements of school closures, incomplete or unavailable public announcements, or lags in data entry. Moreover, lengths of closure may be unknown when the specific date of reopening cannot be ascertained or because the school or district remains closed. Furthermore, learning modality may not be specified in the data abstraction source (the announcement, website, or both) and the data may therefore not capture all transitions from in-person to distance learning. However, the data were collected without burdening schools or districts and were readily available in near real-time. Those limitations probably lead to underestimation of the number and duration of school closures. Therefore, our results likely convey the lower range of the impact of COVID-SCs during this period.

The COVID-SC data we describe were collected as part of an ongoing research project to document how school closures occur outside of an influenza pandemic (*1*). In the absence of a true surveillance system, those data were the most timely and comprehensive data available on COVID-SCs from the early days of the pandemic. This project documented near-simultaneous nationwide closures implemented as a mitigation strategy during the spring of 2020 (*2*) and an unprecedented number of illness-related reactive school closures during the 2 subsequent school years, 2020–21 and 2021–22. These data could be used in conjunction with epidemiologic surveillance and other data for future computer simulations to explore the impact of the COVID-19 pandemic at various geographic levels and to help evaluate effectiveness of contemporaneous pandemic interventions. Specifically, we encourage further research, including high-resolution modeling studies, for locales where early reactive closures occurred (i.e., those with closures occurring in the weeks before COVID-19 peaks were observed in the epidemiologic data) to explore the effects of these closures on communitywide transmission. Given that COVID-19 is already established as an endemic disease and respiratory pathogens other than SARS-CoV-2 will reestablish circulation, local outbreaks of COVID-19, influenza, and other diseases will probably continue to occur, and some will cause reactive school closures. The continued monitoring of disease-related school closures should preserve the ability to detect their occurrence in near–real-time as a component of community-based surveillance during pandemics and severe outbreaks. In addition, ongoing surveillance for disease-related school closures would help in understanding their underlying causes, scale, and distribution and would enable evaluation of their effects on schools and communities.

Appendix Additional information about COVID-19–related school closures, United States, July 27, 2020–June 30, 2022.
