# Supplementary material for: COVID-19–Related School Closures, United States, July 27, 2020–June 30, 2022
Source: Emerg Infect Dis. 2024 Jan;30(1):58–69. doi: 10.3201/eid3001.231215 (PMC10756369; doi:10.3201/eid3001.231215)
Supplement: Appendix — Additional information about COVID-19–related school closures, United States, July 27, 2020–June 30, 2022. [file 23-1215-Techapp-s1.pdf]

Article DOI: https://doi.org/10.3201/eid3001.231215

EID cannot ensure accessibility for supplementary materials supplied by authors. Readers who have difficulty accessing supplementary content should contact the authors for assistance.

COVID-19–Related School Closures, United States, July 27, 2020–June 30, 2022

Appendix

To supplement the methods section, additional information is provided below. Included are methods to calculate the number of school days lost due to COVID-SCs, to conduct descriptive analyses of repeat closures for COVID-19, to describe the number and rate of COVID-SCs by state, and finally to conduct bivariate and multiple logistic regression analysis on school characteristics.

Full List of Inclusion and Exclusion Criteria

Inclusion criteria for schools and school districts:

- Public school or school district, or private school, which:
  - o Provided in-person education;
  - o Were located in the United States (50 states and District of Columbia);
  - o And, served students between kindergarten and twelfth grades inclusive.

Districts were excluded for the following reasons:

- Did not have any schools (ex., administrative districts); or
- Did not have or report student enrollment.

Schools were excluded for the following reasons:

- Did not provide in-person learning:
  - o Permanent distance-learning only schools.
- Did not meet the student grade-span criteria:

o Schools that served only pre-kindergarten and/or transitional kindergarten students;

o Adult education schools.

- Did not have or report student enrollment:

o Schools with zero reported students in NCES databases (CCD and PSS);

o Vocational, special education, and alternative schools with missing values or no information for student enrollment.

- Juvenile justice alternative education program or detention centers.

Description of Reasons for COVID-19–Related School Closures

Reasons for COVID-19-related school closures were abstracted from school and school district closure announcements. These reasons fell into two primary categories, transmission related and non-transmission-related reasons. Transmission-related reasons for closure were those that were specifically attributed to measures of ongoing transmission (ex., cases in schools, community clusters, etc.) or to the effects of ongoing transmission (ex., increased absenteeism, quarantining of students/staff). Though also specific to COVID-19, non-transmission-related reasons for closure were those that were not a direct result or effect of ongoing transmission. These included reasons that would not have occurred in the absence of the COVID-19 pandemic (ex., COVID-19 vaccinations, pandemic-induced mental health days).

List of Transmission-Related Reasons for COVID-SCs:

- Confirmed or reported COVID-19 cases in the school or school district;

- Suspected COVID-19 cases in the school or school district;

- Increased student and/ or staff absenteeism in the school or school district;

- Clusters or widespread transmission in the local community;

- State or local guidance/mandate to close schools in response to COVID-19 transmission in the school, school district, or local community;

- To clean/disinfect facilities or property following known or suspected COVID-19 cases; and

- Other reasons related to COVID-19 mitigation:

  o COVID-19 testing, contact tracing, quarantine of students and/or staff, prevention of holiday-related transmission surge, death of staff member, lack of community resources (ex., contact tracers), and noncompliance with governor's executive orders.

List of Non-transmission-Related Reasons for COVID-SCs:

- COVID-19 vaccinations;

- Side-effects of COVID-19 vaccinations;

- Teacher/staff shortages attributed to hiring and retention challenges (with specific attribution to the COVID-19 pandemic);

- For student/staff mental health days; and

- Other reasons associated with COVID-19:

  o Protests over in-person learning, protests over facemask policies, transportation issues specific to COVID-19, lack of resources specific to COVID-19, and in order for schools and school districts to work on their respective COVID-19 mitigation plans.

Factors Considered when Calculating Correlations

When assessing correlations of COVID-SCs with epidemiologic surveillance data, school winter break was excluded from the analysis because schools and school districts were inherently unable to close for COVID-19-related reasons when they were on a planned break.

Correlations were calculated using three different scenarios for the winter break. First, the final week of the initial calendar year was excluded (epidemiologic week 53 in 2020 and epidemiologic week 52 in 2021). Second, the two finals weeks of the initial calendar year were excluded (weeks 52–53 in 2020 and weeks 51–52 in 2021). Third, the final week of the initial calendar and the first week of the next calendar year were excluded (week 53 of 2020 and week 1 of 2021 for the 2020–21 school year, and week 52 of 2021 and week 1 of 2022 for the 2021–22 school year).

While the break is understood to be approximately 2 weeks in length, the start and end dates vary by school and school district. However, winter breaks consistently overlap on the last week of the calendar year, which was epidemiologic week 53 in 2020 and epidemiologic week 52 in 2021. To reflect this consistency, results reported in the main text represent those excluding only the final weeks of the calendar year. However, results from all three winter break exclusion scenarios are presented in Table 3 (https://wwwnc.cdc.gov/EID/article/30/01/23-1215-T3.htm).

Additional Description of Methods

To supplement the methods section, additional information is provided below. Included are methods to calculate the number of school days lost due to COVID-SCs, to conduct descriptive analyses of repeat closures for COVID-19, to describe the number and rate of COVID-SCs by state, and finally to conduct bivariate and multiple logistic regression analysis on school characteristics.

In-Person School Days Lost due to COVID-SCs

In-person school days lost due to COVID-SCs were calculated by counting the total number of days between the date of closure (inclusive) and the date of reopening (exclusive), and then subtracting any planned closure days such as weekends, planned holidays, or teacher in-service days. The number of COVID-SCs by number of in-person school days lost were charted with whisker plots of the annual distributions of unplanned closure days overlain, using Power BI. Median number of in-person school days lost by state and by year were calculated and mapped using Power BI.

Repeat Closures for COVID-19

During the course of analysis, repeat closures of the same schools for COVID-19 were noted in the data within each school year. We describe the patterns seen across the study period.

Reasons for Closures

In addition to describing reasons for all COVID-SCs by school year, the states with the highest median number of in-person school days lost per closure were described separately.

COVID-SCs by State

Cumulative incidence of COVID-SCs were calculated for the 2021/22 school year, with the number of COVID-SCs being the numerator and the total number of K-12 schools (public [1]

and private [2], as reported by NCES) being the denominator. The number of COVID-SCs and the cumulative incidence of closures were mapped by state for the 2021/22 school year using Power BI.

Bivariate and Multiple Logistic Regression

Bivariate and multivariable logistic regression were performed using PROC LOGISTIC to examine both the unadjusted and adjusted odds ratios between COVID-SCs and certain school characteristics in the NCES dataset for public schools (*1*), which comprise 98.4% of schools in our dataset. Private schools accounted for the remaining 1.6%% of COVID-SCs and were excluded as the NCES Private School Survey data (*2*) did not fully include the corresponding variables found in public school data. The dependent variable was the number of unique schools closed for COVID-19 vs the total number of schools that did not close due to COVID-19, and the independent variables were urbanicity, student-teacher ratio, and percentage of students eligible for free or reduced-price school meals. Analysis was conducted using SAS 9.4 (SAS Institute Inc., Cary, North Carolina).

Supplementary Results

In-Person School Days Lost due to COVID-19–Related School Closures

See Appendix Figures 1 and 2.

Repeat Closures for COVID-19

Repeat closures for COVID-19 were concentrated in HHS 4 for both 2020–2021 and 2021–2022 school year (Appendix Table 1). For 2020–2021 school year, more than one-third of the COVID-19-related repeat closures were observed in suburban areas, while majority of the repeat closure for 2021–2022 school year were concentrated in city areas.

Reasons for Closures

Among the seven states with the highest median number of in-person school days lost per closure in SY 2020–21 (>20 days in California, Colorado, Illinois, Indiana, Kentucky, Minnesota, and Nevada), the most common reasons for both district and school-level closure events was due to cluster or widespread transmission in the community (83.1% and 55.4%, respectively) (Appendix Table 2).

COVID-SCS by State

See Appendix Figure 3.

Regression Analysis

During the 2020–2021 school year, schools located in rural areas (aOR 0.47, 95% CI 0.45–0.50), towns (aOR 0.54, 95% CI 0.51–0.58), or suburban areas (aOR 0.67, 95% CI 0.64–0.71) (Appendix Table 2) had lower odds of experiencing closure than those located in cities. Meanwhile, the chance of school closure was significantly lower for the highest quartile of student-teacher ratio (aOR 0.72, 95% CI 0.68–0.76). Schools in the upper two quartiles of students eligible for free or reduced lunch showed significant lower odds of COVID-SCs as compared to first quartile (Q3: aOR 0.87, 95% CI 0.82–0.92, and Q4: aOR 0.89, 95% CI 0.84–0.94).

Similar to the previous SY, during the 2021–2022 SY, schools located in rural areas (aOR 0.54, 95% CI 0.52–0.57), towns (aOR 0.65, 95% CI 0.61–0.69), or suburban areas (aOR 0.69, 95% CI 0.66–0.72) had lower odds of experiencing a COVID-SC than those located in cities (Appendix Table 2). Schools in the 3rd quartile of student-teacher ratio showed higher odds of closure (aOR 1.25, 95% CI 1.18–1.31) followed by the 2nd quartile (aOR 1.18, 95% CI 1.12–1.24) when compared to the lowest quartile, while schools in the highest quartile of student-teacher ratio showed lower odds (aOR 0.77, 95% CI 0.73, 0.814). The chance of school closures increased as the percentage of students eligible for free or reduced lunch increased, the highest odds of closures were observed in the highest quartile (aOR 1.61, 95% CI 1.53, 1.70) when compared to the lowest quartile (Appendix Table 2). See Appendix Table 3.

References

1. National Center for Education Statistics. Common core of data. [cited 2023 Apr 5]. http://nces.ed.gov/ccd.

2. National Center for Education Statistics. Private School Universe Survey [cited 2022 Mar 21]. https://nces.ed.gov/surveys/pss

3. US Department of Health and Human Services. Regional offices [cited 2023 Mar 7]. https://www.hhs.gov/about/agencies/regional-offices/index.html?language=es

Appendix Table 1. Recurrence of COVID-related school closures among unique schools*– United States, July 27, 2020 – June 30, 2022

Characteristics of schools closed	Unique schools, n (%)	Unique schools by number of closures, n (%)				
		Single closure occurrence, n (%)	Multiple closures, n (%)			
			Total (≥2X)‡	2X	3–4X	≥5X‡
2020–2021 School Year						
Total§	16,890	14,854 (87.9)	2,036 (12.1)	1,756 (10.4)	267 (1.6)	13 (0.1)
Urbanicity						
City	5,997 (35.5)	5,351 (36.0)	646 (31.7)	570 (32.5)	73 (27.3)	3 (23.1)
Suburban	5,297 (31.4)	4,585 (30.9)	712 (35.0)	633 (36.1)	72 (27.0)	7 (53.9)
Town	1,687 (10.0)	1,469 (9.9)	218 (10.7)	174 (9.9)	43 (16.1)	1 (7.7)
Rural	3,630 (21.5)	3,174 (21.4)	456 (22.4)	375 (21.4)	79 (29.6)	2 (15.4)
Not specified	279 (1.7)	275 (1.9)	4 (0.2)	4 (0.2)	0	0
HHS Region¶						
HHS 1	1,049 (6.2)	851 (5.7)	198 (9.7)	154 (8.8)	42 (15.7)	2 (15.4)
HHS 2	2,795 (16.0)	2,319 (15.6)	386 (19.0)	341 (19.4)	44 (16.5)	1 (7.7)
HHS 3	2,399 (14.2)	2,019 (13.6)	380 (18.7)	298 (17.0)	76 (28.5)	6 (46.2)
HHS 4	3,455 (20.5)	2,997 (20.2)	458 (22.5)	407 (23.2)	48 (18.0)	3 (23.1)
HHS 5	3,254 (19.3)	2,910 (19.6)	344 (16.9)	317 (18.1)	27 (10.1)	0
HHS 6	926 (5.5)	821 (5.5)	105 (5.2)	87 (5.0)	18 (6.7)	0
HHS 7	566 (3.4)	544 (3.7)	22 (1.1)	22 (1.3)	0	0
HHS 8	782 (4.6)	694 (4.7)	88 (4.3)	86 (4.9)	2 (0.8)	0
HHS 9	1,418 (8.4)	1,384 (9.3)	34 (1.7)	33 (1.9)	1 (0.4)	0
HHS 10	336 (2.0)	315 (2.1)	21 (1.0)	11 (0.6)	9 (3.4)	1 (7.7)
2021–2022 School Year						
Total§	19,871	15,404 (77.5)	4,467 (22.5)	3,620 (18.2)	686 (3.5)	161 (0.8)
Urbanicity						
City	7,338 (36.9)	4,653 (30.2)	2,685 (60.1)	2,173 (60.0)	430 (62.7)	82 (50.9)
Suburban	5,966 (30.0)	4,948 (32.1)	1,018 (22.8)	753 (20.8)	201 (29.3)	64 (39.8)
Town	2,215 (11.2)	1,996 (13.0)	219 (4.9)	204 (5.6)	9 (1.3)	6 (3.7)
Rural	4,286 (21.6)	3,747 (24.3)	539 (12.1)	484 (13.4)	46 (6.7)	9 (5.6)
Not specified	66 (0.3)	60 (0.4)	6 (0.0)	6 (0.2)	0	0
HHS Region¶						
HHS 1	554 (2.8)	517 (3.4)	37 (0.8)	27 (0.8)	10 (1.5)	0
HHS 2	1,334 (6.7)	1,200 (7.8)	134 (3.0)	131 (3.6)	3 (0.4)	0
HHS 3	2,029 (10.2)	1,534 (10.0)	495 (11.1)	338 (9.3)	152 (22.2)	5 (3.1)
HHS 4	4,726 (23.8)	4,084 (26.5)	642 (14.4)	591 (15.3)	51 (7.4)	0
HHS 5	3,946 (19.9)	2,363 (15.3)	1,583 (35.4)	1,322 (36.5)	227 (33.1)	34 (21.1)
HHS 6	3,362 (16.9)	2,873 (18.7)	489 (11.0)	380 (10.5)	94 (13.7)	15 (9.3)
HHS 7	1,206 (6.1)	918 (6.0)	288 (6.5)	202 (5.6)	68 (9.9)	18 (11.2)
HHS 8	930 (4.7)	482 (3.1)	448 (10.0)	381 (10.5)	9 (1.3)	58 (36.0)
HHS 9	768 (3.9)	727 (4.7)	41 (0.9)	23 (0.6)	0	18 (11.2)
HHS 10	1,016 (5.1)	706 (4.6)	310 (6.9)	225 (6.2)	72 (10.5)	13 (8.1)

*Unique school: each school experiencing closure was counted only once.
†School year: 2020–2021 (July 27, 2020 to June 30, 2021), 2021–2022 (Aug 1, 2021 to Jun 30, 2022).
‡The maximum number of repeat closures was seven times during 2020–2021 school year and eight times during 2021–2022 school year.
§Total row presented with row percent, all else reported with column percent.
¶Regions of the United States Department of Health & Human Services (HHS) (3).

Appendix Table 2. Reasons* for COVID-19 Related K-12 School Closure events– Seven States† (California, Colorado, Illinois, Indiana, Kentucky, Minnesota, and Nevada), July 27, 2020—June 30, 2021

Reasons* for school closure decision stated in announcement of COVID-19-related closure‡	District level§	Individual school level
Number of schools closed due to COVID-19 related closure events	3,209	652
Number of schools closed where closure announcement mentions only COVID-19	40 (1.2)	5 (0.8)
Number of schools closed where closure announcement mentions COVID-19 and specific reasons	3,169 (98.8)	647 (99.2)
·ansmission-related reasons		
Due to positive case(s)	768 (23.9)	290 (44.5)
In student(s)	239 (7.5)	85 (13.0)
In staff member(s)	229 (7.1)	103 (15.8
In household member of student/staff	0 (0.0)	2 (0.3)
Due to suspected case(s)	24 (0.8)	27 (4.1)
In student(s)	11 (0.3)	4 (0.6)
In staff member(s)	8 (0.3)	17 (2.6)
In household member of student/staff		1 (0.2)
Due to increased student absenteeism	110 (3.4)	14 (2.2)
Due to increased staff absenteeism	494 (15.4)	74 (11.4)
Due to cluster or widespread transmission in the community	2,668 (83.1)	361 (55.4)
Due to state or local guidance/mandate	924 (28.8)	200 (30.7)
To clean/disinfect classrooms, buildings, and facilities	47 (1.5)	24 (3.7)
Other ¶	93 (2.9)	75 (11.5)
·Ion-transmission-related reasons		
Vaccination of staff	22 (0.7)	0 (0.0)
Side effects of vaccination	0 (0.0)	1 (0.2)

*Reasons are recorded as stated in the school closure announcements.
†The seven states (California, Colorado, Illinois, Indiana, Kentucky, Minnesota, and Nevada) had the highest median number of in-person schools days lost per closure with a median of >20 d each.
‡Categories are not mutually exclusive because a closure announcement may attribute the closure to more than one factor and/or there may be more than one announcement that contributes the closure to different factors.
§Other reasons include contact tracing, quarantine of students and staff, prevention of holiday-related surge, precautionary measure as a concern of community spread due to union wanting work stoppage, death of staff member, unable to find substitute teachers, transportation issues, critical lack of community resources – including contact tracers, testing, out of an abundance of caution, and flu/other respiratory and/or enteric virus-related illnesses, internet outage, facility issues, and noncompliance with Governor's executive orders.
¶Other reasons include staff protesting in-person learning, protest over mask policy, transportation issue, lack of resources, allowing time for testing, and work on the COVID-19 mitigation plan.

Appendix Table 3. Selected characteristics of COVID-19-related unique* public school closures by school year† — United States, July 27, 2020—June 30, 2022‡

	2020–2021§				2021–2022§			
Characteristic	Unadjusted OR (95% CI)	P	Adjusted OR (95% CI)	P	Unadjusted OR (95% CI)	P	Adjusted OR (95% CI)	P
Urbanicity								
City	Ref.		Ref.		Ref.			
Rural	0.54 (0.51, 0.56)	<0.001	0.47 (0.45, 0.50)	<0.001	0.49 (0.47, 0.51)	<0.001	0.54 (0.52, 0.57)	<0.001
Town	0.55 (0.52, 0.59)	<0.001	0.54 (0.51, 0.58)	<0.001	0.57 (0.54, 0.60)	<0.001	0.65 (0.61, 0.69)	<0.001
Suburban	0.72 (0.69, 0.75)	<0.001	0.67 (0.64, 0.71)	<0.001	0.65 (0.62, 0.67)	<0.001	0.69 (0.66, 0.72)	<0.001
Student-teacher ratio¶								
< = Q1	Ref.		Ref.		Ref.		Ref.	
>Q1-Q2	1.13 (1.07, 1.19)	<0.001	1.06 (1.00, 1.12)	0.040	1.26 (1.21, 1.32)	<0.001	1.18 (1.12, 1.24)	<0.001
>Q2-Q3	1.06 (1.01, 1.11)	<0.001	0.95 (0.90, 1.00)	0.064	1.35 (1.29, 1.42)	<0.001	1.25 (1.18, 1.31)	<0.001
>Q3	0.83 (0.79, 0.87)	<0.001	0.72 (0.68, 0.76)	<0.001	0.91 (0.87, 0.95)	<0.001	0.77 (0.73, 0.81)	<0.001
Percent of students eligible for free or reduced school meals#**								
< = Q1	Ref.		Ref.		Ref.		Ref.	

Characteristic	2020–2021§ Unadjusted OR (95% CI)	P	Adjusted OR (95% CI)	P	2021–2022§ Unadjusted OR (95% CI)	P	Adjusted OR (95% CI)	P
>Q1-Q2	0.91 (0.86, 0.96)	0.003	0.96 (0.91, 1.02)	0.156	1.30 (1.24, 1.37)	<0.001	1.29 (1.23, 1.35)	<0.001
>Q2-Q3	0.90 (0.86, 0.95)	<0.001	0.87 (0.82, 0.92)	<0.001	1.60 (1.53, 1.68)	<0.001	1.47 (1.39, 1.54)	<0.001
>Q3	1.02 (0.97, 1.08)	<0.001	0.89 (0.84, 0.94)	<0.001	2.05 (1.95, 2.16)	<0.001	1.61 (1.53, 1.70)	<0.001

*Unique school: each school experiencing closure was counted only once.
†School year: 2020–2021 (July 27, 2020 to June 30, 2021), 2021–2022 (Aug 1, 2021 to Jun 30, 2022).
‡Bivariate and multivariate logistic regression were used for the unadjusted and adjusted odds ratios respectively. Dependent variable: The total number of unique schools closed during the period vs the total number of schools that didn't close. Independent variables: urbanicity, student-teacher ratio, and percentage of students eligible for free or reduced-price school meals.
§The total number of unique public schools that matched with the NCES public school data [1] and used for this analysis was 16,188 for 2020–2021 school year and 19,763 for 2021–2022 school year.
¶Student-teacher ratio by year: 2020–2021 - The lower quartile (Q1), median (Q2), and upper quartile (Q3) was 12.05, 14,27, and 16.84 respectively. 2021–2022 - The lower quartile (Q1), median (Q2), and upper quartile (Q3) was 12.37, 14,54, and 16.87 respectively.
#Percent of students eligible for free or reduced meals by year: 2020–2021 - The lower quartile (Q1), median (Q2), and upper quartile (Q3) was 32.11, 54.16, and 80.76 respectively. 2021–2022 - The lower quartile (Q1), median (Q2), and upper quartile (Q3) was 36.83, 59.97, and 84.80 respectively.
**Private schools were excluded from this analysis; the data are available for public schools only (*1*).

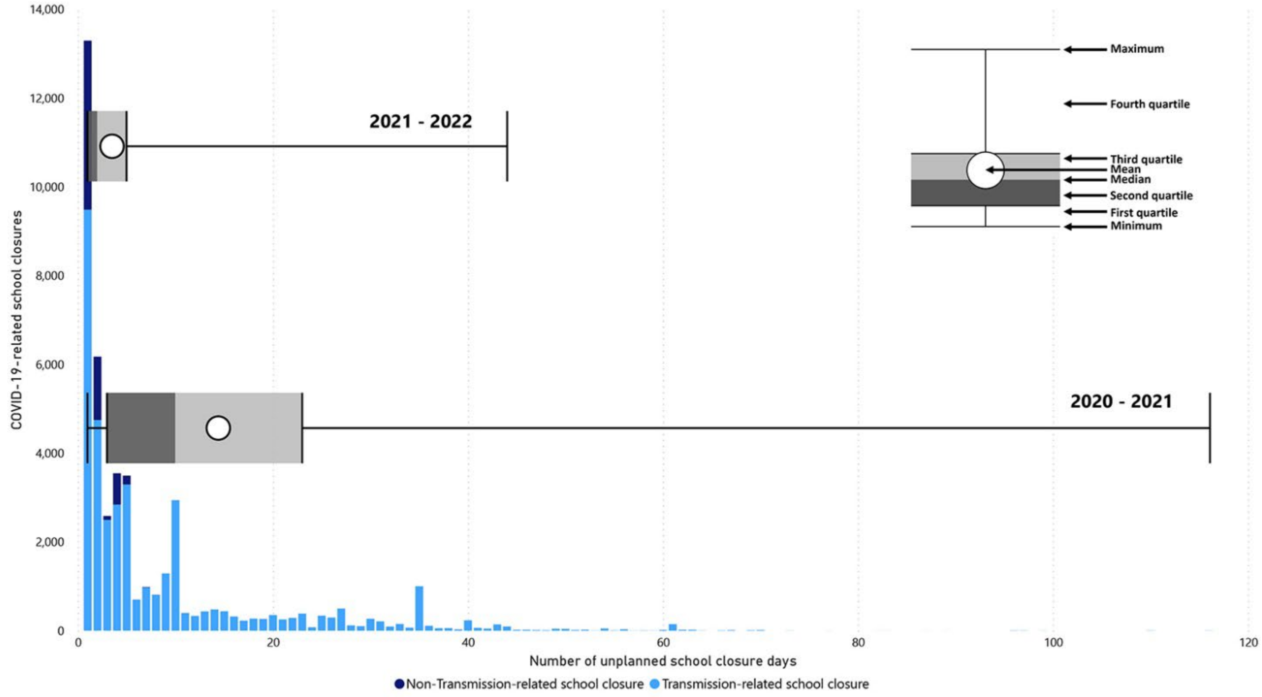

Appendix Figure 1. Number of In-Person School Days Lost Due to COVID-19-related School Closures per Closure by Closure Type, United States, July 27, 2020 – June 30, 2022. In-Person school days lost includes only unplanned closure days and excludes weekends and planned closure days (holidays, teacher workdays, etc.). These are verified using school or school district calendars when available. Academic year 2020 – 2021: median 10, mean 14, range 1-116. Academic year 2021 – 2022: median 2, mean 3, range 1-44. School closure is defined as a transition from being open to being closed for in-person instruction excluding any scheduled days off; fully in-person and hybrid learning modalities are classified as open, and fully remote and closed are classified as closed. Transmission-related reasons

include COVID-19 cases, suspected cases, increased student absenteeism, increased staff absenteeism, cluster or widespread transmission in the community, state or local guidance/mandate to close schools in response to COVID-19, to clean/disinfect school facilities, and other. Non-transmission-related reasons include COVID-19 vaccinations and side effects of vaccination of staff/students, teacher/staff shortage, for student/staff mental health, and other reasons associated with COVID-19.

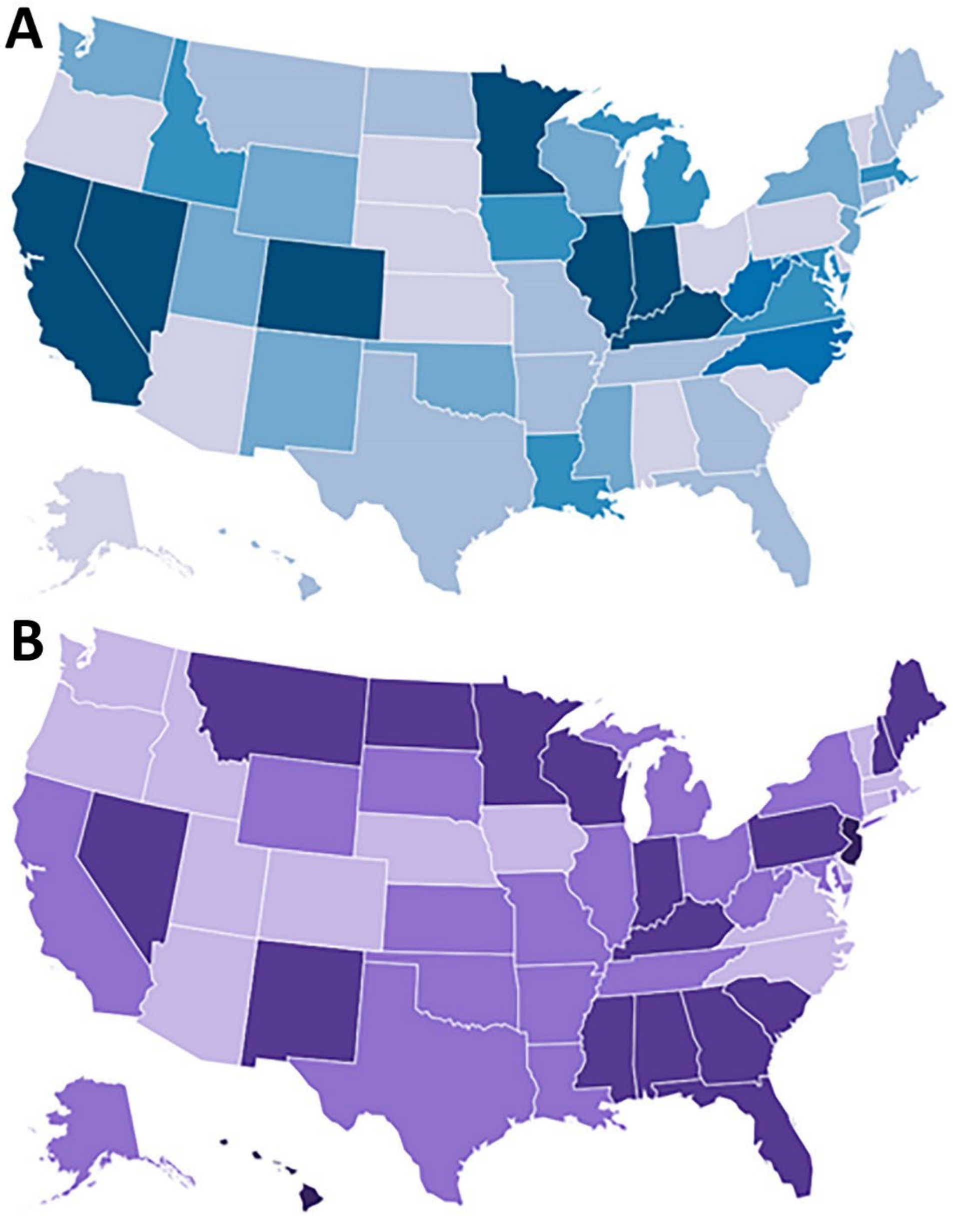

Appendix Figure 2. Median number of in-person school days lost due to COVID-19-related school closure by state, United States, July 27, 2020 - June 30, 2022. School closure is defined as a transition from being open to being closed for in-person instruction, excluding any scheduled days off. Fully in-

person and hybrid learning modalities are classified as open, and fully remote and closed are classified as closed. In-person school days lost includes only unplanned closure days and excludes weekends and planned closure days (holidays, teacher workdays, etc.). These are verified using school or school district calendars when available. Transmission-related reasons include COVID-19 cases, suspected cases, increased student absenteeism, increased staff absenteeism, cluster or widespread transmission in the community, state or local guidance/mandate to close schools in response to COVID-19, to clean/disinfect school facilities, and other. Non-transmission-related reasons include COVID-19 vaccinations and side effects of vaccination of staff/students, teacher/staff shortage, for student/staff mental health, and other reasons associated with COVID-19.

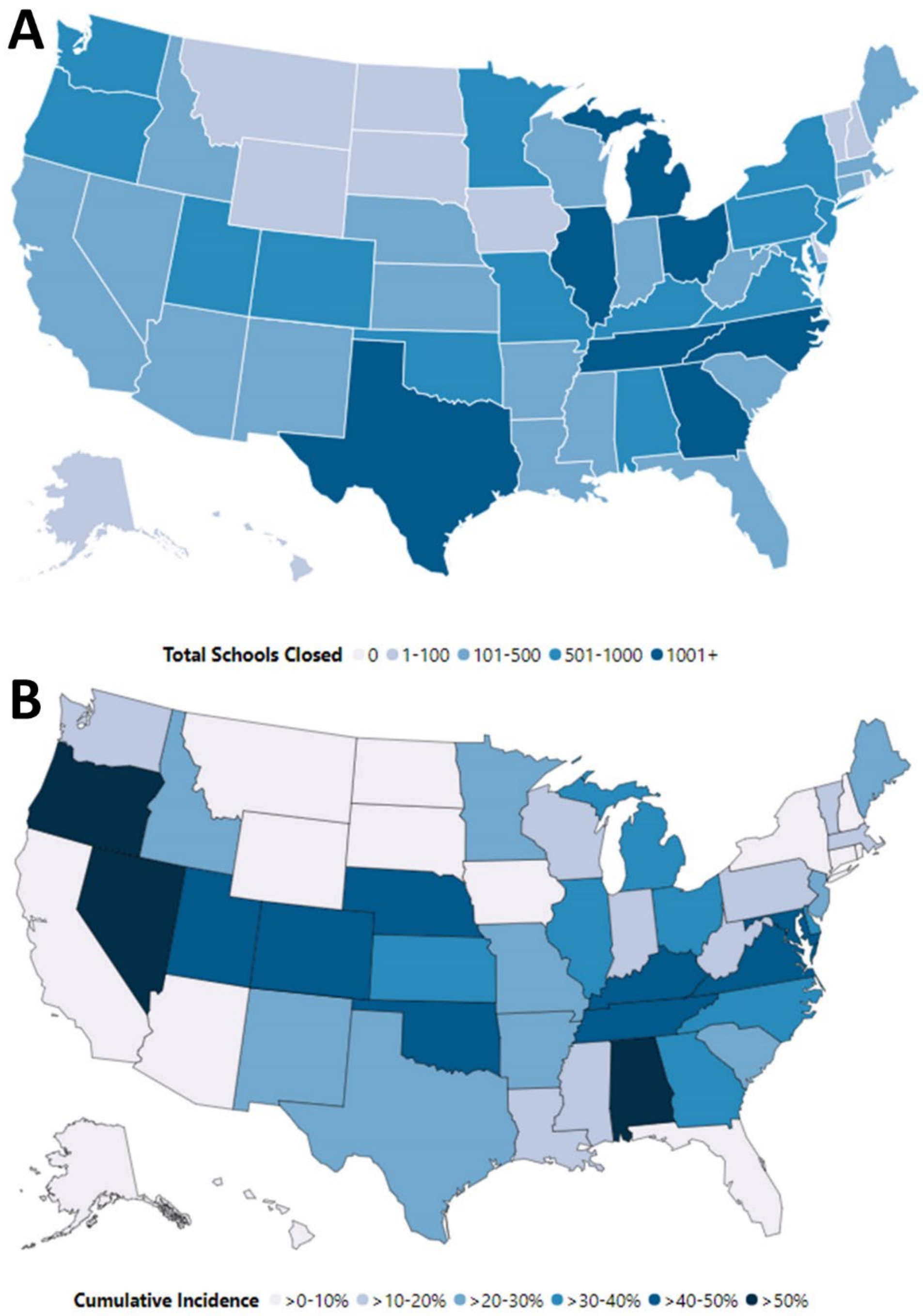

Appendix Figure 3. Number of COVID-19-related school closures and cumulative incidence of closures by states, United States, August 1, 2021 – June 30, 2022. School closure is defined as a transition from being open to being closed for in-person instruction excluding any scheduled days off; fully in-person and hybrid learning modalities are classified as open, and fully remote and closed are classified as closed. Transmission-related reasons include COVID-19 cases, suspected cases, increased student absenteeism, increased staff absenteeism, cluster or widespread transmission in the community, state or local guidance/mandate to close schools in response to COVID-19, to clean/disinfect school facilities, and

other. Non-transmission-related reasons include COVID-19 vaccinations and side effects of vaccination of staff/students, teacher/staff shortage, for student/staff mental health, and other reasons associated with COVID-19. The denominator was the total number of k-12 schools (public &private) in 2021 NCES databases (*1*,*2*).
